# Lymphoproliferative malignancies in patients with neurofibromatosis 1

**DOI:** 10.1186/s13023-021-01856-8

**Published:** 2021-05-19

**Authors:** Christina Bergqvist, François Hemery, Arnaud Jannic, Salah Ferkal, Pierre Wolkenstein

**Affiliations:** 1 Faculty of Medicine, Université Paris-Est Creteil (UPEC), 94010 Créteil, France; 2 Service de Dermatologie, Assistance Publique-Hôpital Paris (AP-HP), Hôpital Henri-Mondor, 94010 Créteil, France; 3 Department of Medical Informatics, Hôpital Henri-Mondor, AP-HP, 94010 Créteil, France; 4 INSERM, Centre d’Investigation Clinique 006, Assistance Publique-Hôpital Paris (AP-HP), Hôpital Henri-Mondor, Referral Center of Neurofibromatoses, 94010 Créteil, France; 5 Faculty of Medicine of Paris East Créteil, Department of Dermatology, French Referral Center for Neurofibromatoses, Henri-Mondor Hospital, APHP, University Paris Est Créteil, 94010 Créteil Cedex, France

**Keywords:** Neurofibromatosis 1, Lymphoproliferative diseases, Malignancy, Lymphoma, Leukemia

## Abstract

Neurofibromatosis 1 (NF1) is an inherited, autosomal-dominant, tumor predisposition syndrome with a birth incidence as high as 1:2000. A patient with NF1 is four to five times more likely to develop a malignancy as compared to the general population. The number of epidemiologic studies on lymphoproliferative malignancies in patients with NF1 is limited. The aim of this study was to determine the incidence rate of lymphoproliferative malignancies (lymphoma and leukemia) in NF1 patients followed in our referral center for neurofibromatoses. We used the Informatics for Integrated Biology and the Bedside (i2b2) platform to extract information from the hospital’s electronic health records. We performed a keyword search on clinical notes generated between Jan/01/2014 and May/11/2020 for patients aged 18 years or older. A total of 1507 patients with confirmed NF1 patients aged 18 years and above were identified (mean age 39.2 years; 57% women). The total number of person-years in follow-up was 57,736 (men, 24,327 years; women, 33,409 years). Mean length of follow-up was 38.3 years (median, 36 years). A total of 13 patients had a medical history of either lymphoma or leukemia, yielding an overall incidence rate of 22.5 per 100,000 (0.000225, 95% confidence interval (CI) 0.000223–0.000227). This incidence is similar to that of the general population in France (standardized incidence ratio 1.07, 95% CI 0.60–1.79). Four patients had a medical history leukemia and 9 patients had a medical history of lymphoma of which 7 had non-Hodgkin lymphoma, and 2 had Hodgkin lymphoma. Our results show that adults with NF1 do not have an increased tendency to develop lymphoproliferative malignancies, in contrast to the general increased risk of malignancy. While our results are consistent with the recent population-based study in Finland, they are in contrast with the larger population-based study in England whereby NF1 individuals were found to be 3 times more likely to develop both non-Hodgkin lymphoma and lymphocytic leukemia. Large-scale epidemiological studies based on nationwide data sets are thus needed to confirm our findings.

Dear Editor,

Neurofibromatosis 1 (NF1) is an inherited, fully penetrant autosomal-dominant, tumor predisposition syndrome with a birth incidence as high as 1:2000 [1, 2]. It is caused by mutations in the *NF1* gene on chromosome 17 which encodes neurofibromin, a tumor suppressor protein [3, 4]. NF1 affects multiple systems with cutaneous, neurologic, and orthopedic as major manifestations which lead to significant morbidity or mortality. Major skin features include café-au-lait macules, skinfold freckles and neurofibromas [2]. NF1 is associated with an increased risk of malignancy and a life expectancy about 15 years shorter than the general population [5, 6]. A patient with NF1 is four to five times more likely to develop a malignancy as compared to the general population [7, 8]. Malignancies include malignant brain tumors, malignant peripheral nerve sheath tumors, endocrine cancers, as well as early-onset breast cancer [2]. Over 30 cases of lymphomas in patients with NF1 have been reported to date [9]; however, the number of epidemiologic studies on lymphoproliferative malignancies in patients with NF1 is limited (Table 1). A national study performed in England on 8003 individuals with neurofibromatosis found that NF1 patients were 3 times more likely to develop non-Hodgkin's lymphoma and 7 times more likely to develop chronic myeloid leukemia as compared to the general population [7]. On the other hand, a population-based study in Finland on 1404 NF1 patients did not show a significant increased risk of lymphoid malignancies in NF1 patients [8]. Therefore, the aim of this study was to determine the incidence rate of lymphoproliferative malignancies (lymphoma and leukemia) in NF1 patients followed in our referral center for neurofibromatoses in order to assess the risk in France.

**Table 1:** Relative risks (RR) of lymphoproliferative malignancies in patients with NF1 in the literature

Study	Year	n	Lymphoma RR (95% CI)	Lymphocytic leukemia RR (95% CI)	Studied population
Narod et al. [13]	1991	90	5.1 HL 8.0 NHL 3.4	2.7	Children under the age of 15 years
Stiller et al. [14]	1994	26	NHL 10.0 (3.3–23.4)	5.4 (2.8–9.4)	Children under the age of 15 years
Seminog et al. [7]	2012	8003	NHL 3.3 (1.7–6.0)	2.5 (1.1–4.6)	All age groups
Uusitalo et al. [8]	2016	1404	1.19 (0.39–2.78)		All age groups

*HL* hodgkin lymphoma, *NHL* non-Hodgkin lymphoma

We used the Informatics for Integrated Biology and the Bedside (i2b2) platform to extract information from the hospital’s electronic health records (EHR). We performed a keyword search on clinical notes generated between Jan/01/2014 and May/11/2020 for patients aged 18 years or older. Patients with NF1 were identified using the keyword “Neurofibromatosis”. The visit notes were then read thoroughly, and only patients with a confirmed NF1 diagnosis according to the National Institutes of Health criteria and/or mutation analysis were included [10]. To identify NF1 patients with lymphoproliferative malignancies, each of the following keywords were entered using the Query Tool and an “and” relationship with Neurofibromatosis: “Lymphoma”, “Hodgkin lymphoma”, “Non-Hodgkin lymphoma”, “Diffuse large B cell lymphoma”, “Lymphoblastic lymphoma”, “MALT lymphoma”, “Burkitt lymphoma”, “Marginal zone lymphoma”, “Anaplastic large cell lymphoma”, “Mycosis Fungoides”, “Sezary syndrome”, “Leukemia”, “Chronic lymphocytic leukemia”, “acute lymphocytic leukemia”. Patients with NF1 were observed for these malignancies beginning with their birthdate and ending at death or at the date of their last follow-up, whichever occurred first. The dates of last follow-up or death were obtained using i2b2. Visit notes were then read to determine the age at diagnosis, disease status at last follow-up visit and the presence of another malignancy in the medical history. Incidence rates of lymphomas and leukemias for the general population in France were obtained from the latest study published by the French cancer register in 2019 [11]. Standardized incidence ratios (SIRs) were calculated as the ratios of observed cases and expected cases. Expected cases were obtained by multiplying the person-years with the corresponding population incidence rate. The 95% confidence intervals (CI) were based on the assumption that the number of observed cases followed a Poisson distribution. The study was approved by the national institute concerning health data (INDS) and conducted according to local standards and laws.

We found a total of 2215 patients with the term “Neurofibromatosis” written in their clinical notes. After reading each visit note thoroughly, a total of 1507 patients with confirmed NF1 aged 18 years and above were identified (mean age 39.2 years; range 18–88 years, 57% women). The total number of person-years in follow-up was 57,736 (men, 24,327 years; women, 33,409 years). Mean length of follow-up was 38.3 years (median, 36 years).

A total of 13 patients had a medical history of either lymphoma or leukemia, yielding an overall incidence rate of 22.5 per 100,000 (0.000225, 95% CI 0.000223–0.000227). This incidence rate is similar to that of the general population in France (SIR 1.07, 95% CI 0.60–1.79), indicating that patients with NF1 in this study do not have an increased risk of developing either lymphoma or leukemia. A total of 9 patients had a medical history of lymphoma (Table 2). Seven patients had non-Hodgkin lymphoma and two patients had Hodgkin lymphoma. Four patients had a medical history of leukemia. Three patients had lymphocytic leukemia and one patient had acute myeloid leukemia (Table 2). None of the patients had a history of juvenile myelomonocytic leukemia (JMML). SIRs for each lymphoproliferative disease are found in Table 3.

**Table 2:** Lymphoproliferative malignancies associated with NF1 individuals in our referral center for neurofibromatosis

Individual	Sex	Lymphoproliferative malignancy	Age at diagnosis	Status at last follow up visit	Other malignancy	Diagnosis of other malignancy
*Lymphoma*						
1	F	Hodgkin lymphoma	30	In remission	None	
2	M	Splenic marginal zone lymphoma	68	In remission	Colon adenocarcinoma	Concurrent to lymphoma diagnosis
3	F	Gastric MALT lymphoma	46	In remission	Breast cancer	Two years prior to lymphoma diagnosis
4	M	NK/T lymphoma	17	In remission	Malignant peripheral nerve sheath tumor	After lymphoma diagnosis
5	M	Lymphoblastic lymphoma B	47	On treatment	None	
6	M	Diffuse large B-cell lymphoma	49	In remission	None	
7	M	Splenic marginal zone lymphoma	47	In remission	None	
8	F	Hodgkin lymphoma	NA	NA	None	
9	M	Follicular lymphoma	52	In remission	None	
*Lymphocytic leukemia*						
10	F	Chronic lymphocytic leukemia	67	Stable	Breast cancer	Sixteen years prior to leukemia diagnosis
11	F	Acute lymphoblastic leukemia	5	In remission	None	
12	F	Acute lymphoblastic leukemia	32	On treatment	None	
*Myeloid leukemia*						
13	F	Acute myelogenous leukemia	29	On treatment	Pheochromocytoma	Prior to leukemia diagnosis

*NA* not available, *M* male, *F* female, *MALT* mucosa-associated lymphoid tissue

**Table 3:** Observed and expected numbers of lymphoproliferative diseases and SIRs with 95% CIs among NF1 individuals in our referral center for Neurofibromatoses

Lymphoproliferative disease	Observed	Expected	SIR	95% CI	*p* value
All	13	12.1	1.07	0.60–1.79	0.796
Lymphoma	9	7.8	1.15	0.56–2.12	0.667
Non-Hodgkin lymphoma	7	6	1.17	0.51–2.31	0.683
Hodgkin Lymphoma	2	1.8	1.11	0.19–3.67	0.881
Leukemia	4	4.3	0.93	0.30–2.24	0.885

*SIR* standardized incidence ratios, *CI* confidence interval

None of the patients had an immunosuppressive disorder. Five patients had a medical history of another malignancy; three were prior to, two were concurrent to, and one was after the diagnosis of the lymphoma/leukemia respectively. Individual 3 had breast cancer two years prior to the diagnosis of gastric mucosa-associated lymphoid tissue (MALT) lymphoma; she had been treated with lumpectomy and radiation therapy. Individual 10 had breast cancer sixteen years prior to the diagnosis of chronic lymphocytic leukemia. She had received six cycles of fluorouracil, epirubicin, and cyclophosphamide (FEC-75) and five years of the aromatase inhibitor anastrozole.

Very few studies have evaluated the risk of lymphoproliferative diseases in the NF1 population.

Bader et al. [12] had initially reported in 1978 an increased proportion of mainly nonlymphocytic leukemia among children with NF1, with a marked excess of JMML. Narod et al. [13] were the first to report an increased risk of lymphomas in children with NF1 (Table 1). They had reviewed the records of 16,564 cases of childhood cancer which were reported to the National Registry of Childhood Tumors in Great Britain and found 90 patients with NF1. NF1 children were 8 times more likely to develop Hodgkin’s lymphoma, 3 times more likely to develop non-Hodgkin lymphoma and 3 times more likely to develop acute lymphocytic leukemia. Stiller et al. [14] were the first to carry out a detailed population-based study of leukemia and non-Hodgkin lymphoma associated with NF1. They found that children with NF1 were 10 times more likely to develop non-Hodgkin lymphoma and 5 times more likely to develop acute lymphocytic leukemia compared to the general population. Seminog and Goldacre [7] were the first to perform a population-based study using a linked data set of hospital admissions and deaths in England. They included a large sample size of 8,003 individuals with neurofibromatosis (both NF1 and NF2) of all age groups. These patients were found to be 3 times more likely to develop both non-Hodgkin lymphoma and lymphocytic leukemia. However, their reference population comprised people hospitalized with a range of medical conditions, surgical procedures and injuries, which might not have been representative of the general population. On the other hand, Uusitalo et al. performed a population-based registry in Finland using their national population register center which included 1404 NF1 patients (19,076 person-years) [8, 15]. The incidence of leukemias or lymphomas was not increased in their study. Similarly, adults with NF1 in our referral center for neurofibromatoses did not have an increased tendency to develop either leukemias or lymphoma. Nevertheless, it is possible that the small sample size of our study might have led to results not sufficiently powered to detect such a rare association if present.

*NF1* is a tumor suppressor gene, and the majority of NF1-associated tumors exhibit biallelic inactivation of *NF1* [16]. Neurofibromin functions as a Ras‐GTPase activating protein, and *NF1*mutations lead to over-activation of the Ras signaling pathway [2]. The loss of neurofibromin promotes Ras activity leading to constitutive downstream signaling and increased uncontrolled cell growth. Mutations of the *NF1* gene are frequently found in cancers of the general population, especially in glioblastomas, melanomas, and lung tumors [17]. For examples, somatic *NF1* mutations have been found in 27% of T cell acute lymphoblastic leukemia; however, only 12% were non-synonymous mutations [18]. Very few studies have investigated the mechanism of development of lymphoma and leukemia in patients with NF1. Myeloid leukemic cells from NF1 patients were found to have loss of heterozygosity for the *NF1* gene [19], but in contrast to many non-NF1-associated myeloid malignancies, these cells did not have activating Ras mutations [20]. Furthermore, increased levels of Ras-GTP were reported in the NF1-associated leukemias [21].

An important strength of our study is that all patients had a definitive diagnosis of NF1. An important limitation is that this study is a retrospective study based on the EHR of our referral center for neurofibromatoses; this hospital-based recruitment may have led to a selection bias, as patients with a lymphoproliferative malignancy and/or more severe forms of NF1 are more prone to seek medical care in a referral center and, therefore, may lead to an overestimation of the incidence of lymphoproliferative malignancies. A further limitation is that of left-truncation bias: since our study only included NF1 adults, NF1 children with a lymphoproliferative malignancy such as JMML may have died and thus were not available for study. This may have led to an underestimation of the incidence of lymphoproliferative malignancies. In spite of these potential biases our findings are consistent with the Finish report i.e. an absence of increased risk.

To conclude, our results show that adults with NF1 do not have an increased tendency to develop lymphoproliferative malignancies in their lifetime, in contrast to the general increased risk of malignancy. Large-scale epidemiological studies based on nationwide data sets are needed to confirm our findings.

## Data Availability

Data sharing not applicable to this article as no datasets were generated or analyzed during the current study.
